# Feline Parvovirus Lethal Outbreak in a Group of Adult Cohabiting Domestic Cats

**DOI:** 10.3390/pathogens12060822

**Published:** 2023-06-11

**Authors:** Maria Irene Pacini, Mario Forzan, Giovanni Franzo, Claudia Maria Tucciarone, Milena Fornai, Fabrizio Bertelloni, Micaela Sgorbini, Carlo Cantile, Maurizio Mazzei

**Affiliations:** 1 Department of Veterinary Sciences, University of Pisa, Viale delle Piagge 2, 56124 Pisa, Italy; mario.forzan@unipi.it (M.F.); fabrizio.bertelloni@unipi.it (F.B.); micaela.sgorbini@unipi.it (M.S.); carlo.cantile@unipi.it (C.C.); maurizio.mazzei@unipi.it (M.M.); 2 Department of Animal Medicine, Production and Health (MAPS), University of Padua, Viale dell’Università 16, 35020 Legnaro, Italy; giovanni.franzo@unipd.it (G.F.); claudiamaria.tucciarone@unipd.it (C.M.T.); 3 Ambulatorio Veterinario Piombinese, Via Torino 38, 57025 Piombino, Italy; mile77@inwind.it

**Keywords:** *Felis catus*, feline panleukopenia virus, Protoparvovirus carnivoran1, lethal outbreak

## Abstract

Feline panleukopenia is a highly contagious and often fatal disease in cats. The virus, known as feline panleukopenia virus (FPV), primarily affects kittens and unvaccinated cats. It is transmitted through contact with infected cats or their bodily fluids, as well as contaminated objects and environments. The diagnosis of FPV infection can be confirmed through a combination of clinical signs, blood tests, and fecal testing. Prevention through vaccination is recommended for all cats. This case report describes an outbreak of feline panleukopenia in a group of unvaccinated domestic cats that resulted in acute mortality. The lesions were evaluated using histopathology, and the specific viral strain was characterized using molecular techniques. The clinical course of the outbreak was peracute, with a hemorrhagic pattern and 100% of lethality. The observed clinical-pathological pattern was unusual; nevertheless, molecular studies did not highlight peculiar genomic features of the parvovirus isolate. The outbreak affected 3 out of 12 cats in a very short time. However, the prompt application of biosecurity measures and vaccination resulted in an effective interruption of virus spread. In conclusion, we could assume that the virus found the ideal conditions to infect and replicate at high titers, resulting in a particularly aggressive outbreak.

## 1. Introduction

Feline panleukopenia (FPL) is a worldwide highly contagious disease of Felidae caused by infection with Protoparvovirus carnivoran1 (https://ictv.global/taxonomy; accessed on 8 June 2023), a nonenveloped, single-stranded DNA viral species in the genus *Protoparvovirus*, which includes canine parvovirus and feline panleukopenia virus [1]. Feline panleukopenia virus (FPV) is the major cause of feline panleukopenia, but canine parvovirus (CPV) can also infect and replicate in cats, although rarely [2,3]. FPV has a specific tropism for cells with high mitotic activity, such as bone marrow, lymphoid tissue, and intestinal crypt cells [4]. Viral infection can cause a wide range of signs depending on the virulence of the viral strain, the health status of the host, and the presence of coinfection, but the most common clinical signs are lethargy, anorexia, diarrhea, immunosuppression and often vomiting. In addition, infections acquired during pregnancy may result in abortion or kittens affected by the central nervous system and ocular defects, such as cerebellar hypoplasia, hydrocephalus, retinal dysplasia, and optic nerve hypoplasia [2,5]. The hemogram characterized by severe leukopenia with neutropenia and lymphopenia is considered a diagnostic feature when associated with typical clinical signs [3,5]. The course of the disease is often severe, with an estimated mortality rate from 25% to 100% [6,7]. Due to its high environmental resistance, the virus can remain infectious for months, and disinfection is a key factor in preventing disease transmission in environments with high animal density such as multi-cat households and animal shelters [4,8,9]. Current guidelines recommend vaccination against FPV for domestic cats [10]. 

Because either natural infection or vaccination is common in the population, the identification of FPV-specific antibodies through serological diagnosis is almost inconclusive, while routine diagnosis is based on virus detection [11,12,13]. Viral antigens can be detected in the feces of cats with diarrhea or in secretions and excretions using various commercially available ELISA tests. Direct diagnosis can also be performed via virus isolation or nucleic acid detection using PCR [12,13]. In a small percentage of cases of feline panleukopenia disease, the causative agent is canine parvovirus (CPV), and the correct virus identification is only possible with molecular analysis [14,15]. Despite the widespread vaccination program for FPV, feline panleukopenia remains an important disease, with a high prevalence in the feline population, often with features of severe pathogenicity [2,16,17,18]. However, few data are available on the role of FPV strains in determining the disease outcome or severity [19]. In this study, we describe an outbreak of feline panleukopenia in a group of unvaccinated domestic cats that resulted in acute mortality. The lesions were evaluated using histopathology, and the specific viral strain was characterized using molecular techniques.

## 2. Case Report

The outbreak occurred in a group of 12 cohabiting adult common European cats living in the countryside of the province of Leghorn, central Italy (Table 1). The cats, although domestic, had an outdoor life, roaming freely in the surrounding rural area, and were unvaccinated. In May 2021, an adult female cat was admitted to a private first-aid veterinary clinic, unconscious, with severe hemorrhagic diarrhea and vomiting. The cat died a few hours after the admission. 

The following day, a female cat from the same cohort showed subcorneal hemorrhage, and developed melena, hematemesis, and nasal bleeding 24 h after the first clinical signs. The animal died within 1 day. Due to the peracute status and the rapid progression of clinical signs, it was not possible to perform hematological and biochemical analysis, or diagnostic imaging on the first and second cats.

Two days after the second case, a third male cat was hospitalized with anorexia and depression, and 24 h after the first clinical signs, the cat presented severe abdominal pain, intestinal bleeding, and vomiting. Thus, an abdominal X-ray assessment was performed showing dilated and air-filled intestinal loops. The next day, hypothermia and blindness were also present. The cat died two days after the first clinical signs. 

During hospitalization, a fluid resuscitation protocol was performed on all three cats with one or multiple 20 mL/kg boli of lactated Ringer’s solution (LRS). Then, a continuous rate of infusion of LRS was maintained throughout the entire time of hospitalization [20]. 

All three cats tested negative for parvovirus and coronavirus in point-of-care (POC) tests (Theratest Parvo/Corona, Bioforlife, Italy; Test SNAP Parvo, IDEXX, Westbrook, ME, USA) applied on fecal samples collected during the first clinical examination, and also tested negative for FIV and FeLV (SNAP FIV/FeLV Combo Test, IDEXX, USA) on whole-blood samples. Blood work revealed severe anemia, panleukopenia, and thrombocytopenia, as well as hypoalbuminemia and hypoproteinemia.

The second and third deceased cats (named cat #1 and cat #2) were sent to the Department of Veterinary Sciences (DSV) of Pisa for anatomopathological, bacteriological, and virological investigations. 

In the hypothesis of an infectious cause, the remaining healthy cats (aged 1 to 12 years) were isolated to limit the spread of the disease. The objects that had been in contact with the cats were destroyed, and surfaces were thoroughly cleaned and disinfected with 1% sodium hypochlorite. Finally, core vaccination was administered (two doses at an interval of 4 weeks) to all the surviving cats, and no further clinical cases were reported.

### 2.1. Anatomopathological Examination

**Figure 1:**
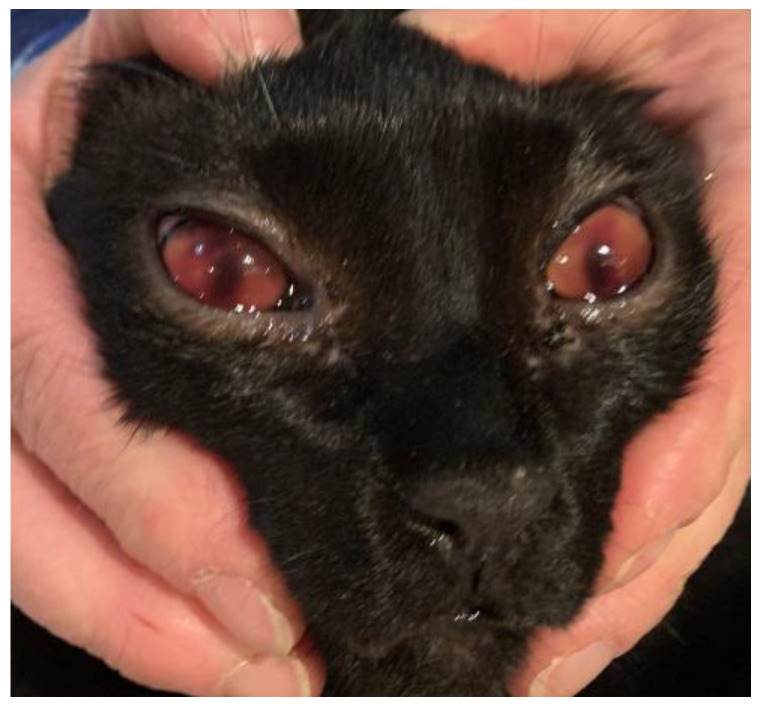
Cat #1. Bilateral hyphema.

**Figure 2:**
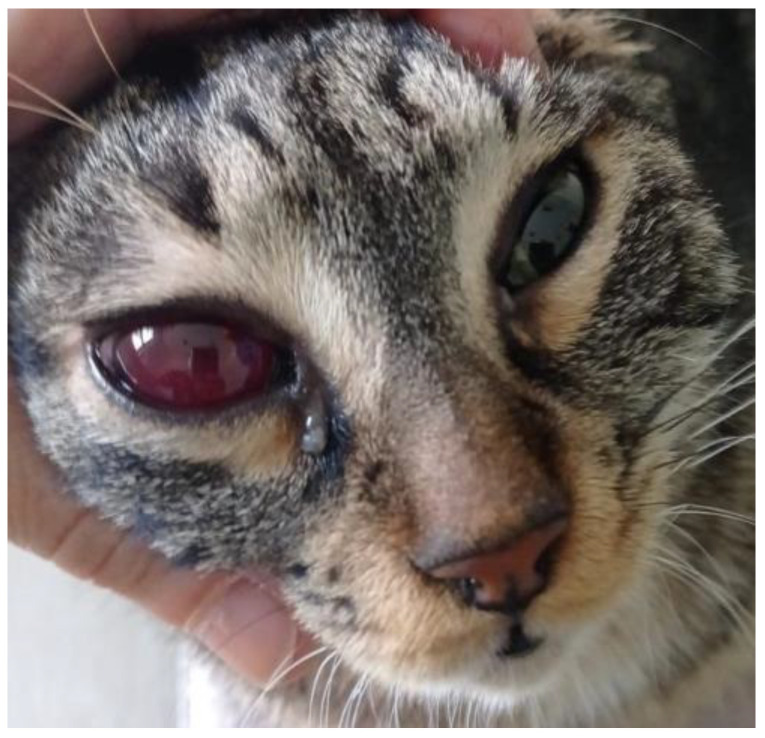
Cat #2. Right hyphema.

**Figure 3:**
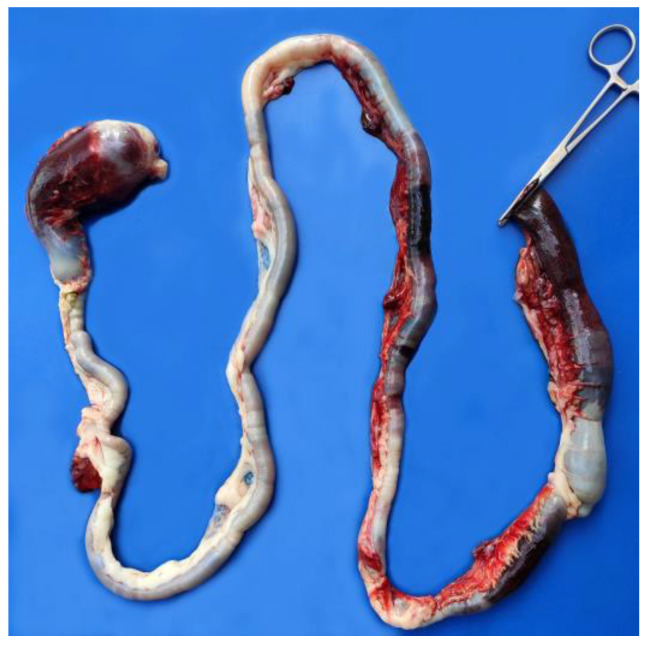
Cat #1. Severe and extensive gastrointestinal infarctions.

**Figure 4:**
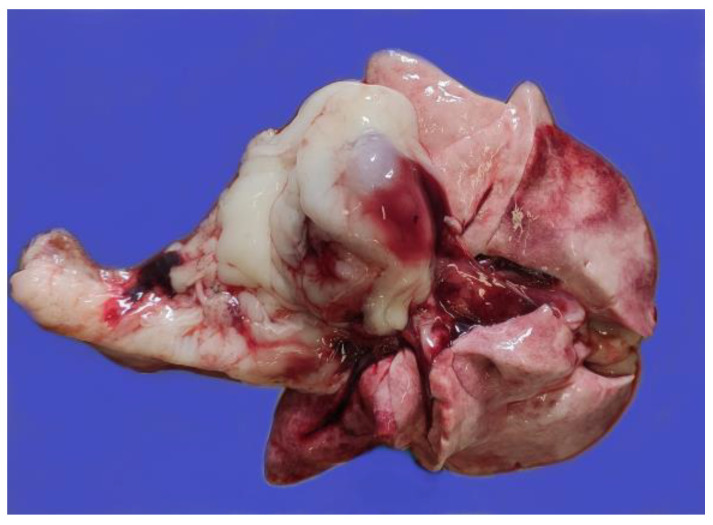
Cat #2. Hemorrhage in the mediastinum and right apical pulmonary lobe.

The postmortem examination of the cats revealed hemorrhagic fecal soiling of the perineal area and severe bilateral hyphema in cat #1 (Figure 1) and right eye hyphema in cat #2 (Figure 2). Both cats showed severe anemia with mild serum–blood effusions in the peritoneal and pleural cavities and mild discoloration of the liver and kidneys. In both cats, but more extensively in cat #1, the gastrointestinal tract showed severe multiple segmental hemorrhages with the transmural involvement of large portions of the stomach, small intestine, and colon (Figure 3), with hemorrhage also involving the mesenteric perivascular tissue and lymph nodes. The intestine contained bloody liquid material. These lesions were interpreted as multiple gastrointestinal infarctions. The bone marrow of cat #1 was pale and gelatinous. Minor hemorrhages were observed in the mediastinum, right chest wall, and left apical pulmonary lobe of cat #2 (Figure 4). 

Samples for histopathological examination were collected from all major organs, including ocular globes. Globes were fixed in Davidson’s solution, while other tissue samples were fixed in 10% neutral buffered formalin. The fixed tissues were then routinely processed for histology and 5 μm thick sections were stained with hematoxylin and eosin and Masson’s trichrome stain.

**Figure 5:**
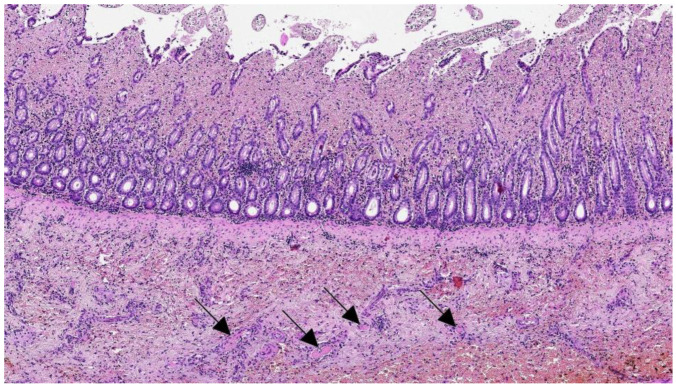
Cat #1, ileum. Severe necrohemorrhagic enteritis with intravascular coagulation within the submucosal vessels (arrows) (HE, 80×).

**Figure 6:**
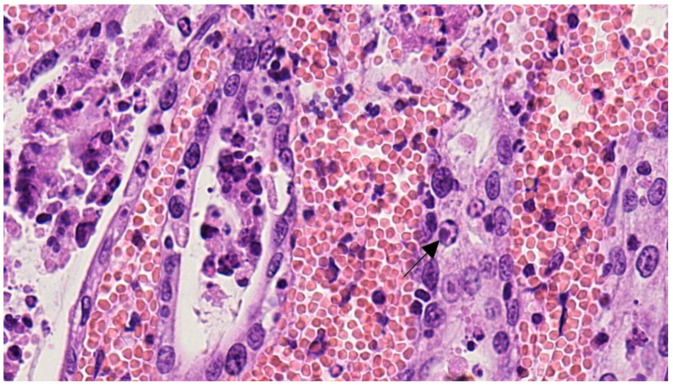
Cat #1, ileum. Crypts containing sloughing epithelial cells with amphophilic intranuclear inclusion bodies (arrow) (HE, 400×).

Histopathological examination of the stomach, ileum, and colon showed severe diffuse transmural hemorrhages, with widespread deposition of fibrin and platelets in small gastrointestinal vessels. Numerous granulocytes, lymphocytes, and monocytes infiltrated the mucosa, submucosa, and muscle layer, in the presence of edema, congestion, and hemorrhage (Figure 5). In the small intestine, the inflammatory infiltration of the lamina propria of the villi was associated with crypt dilation and necrosis, as well as the occasional presence of intranuclear bodies (Figure 6). In the kidneys, glomerular thrombocapillaritis was observed in the presence of acellular protein material in the Bowman’s space. In the lungs, multifocal peribronchiolar hemorrhage was observed with diffuse acute capillary congestion. Macrophage accumulation with erythrophagocytosis was noted in the cortical sinuses of the mesenteric lymph nodes. No significant lesions were observed in other tissues. 

### 2.2. Bacteriology

Lung, spleen, ileum, and colon samples were homogenized in sterile saline water using a Stomacher^®^ 80 Biomaster instrument. A loopful of each homogenate was stretched on Columbia Blood Agar plates (Oxoid, Basingstoke, UK) and incubated at 37 °C under aerobic and anaerobic conditions for up to 10 days. Plates were checked daily for bacterial growth, and no pathogenic bacteria were detected in the analyzed samples.

### 2.3. Virology

#### 2.3.1. Viral Isolation 

**Figure 7:**
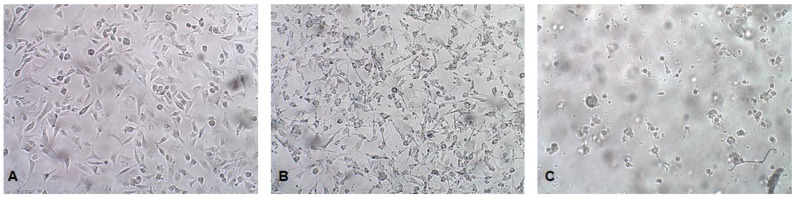
Virus isolation: CRFK cells were infected at the mitotic phase, and pictures were taken with an inverted microscope at 40× magnification: (**A**) mock (72 h); (**B**) 48 h p.i.; (**C**) 72 h p.i.

Tissues collected from cat #1 during necroscopy were transferred to a homogenizer bag in the presence of 1.5 mL of serum-free MEM containing 1% penicillin–streptomycin. The tissues were homogenized in a Stomacher^®^ 80 Biomaster using 4 min high-frequency strokes, and then 1 mL of medium was collected and transferred to a 2 mL sterile tube. Cellular debris was pelleted via centrifugation at 8000 rpm for 5 min. The medium was finally collected and used to infect CRFK cells in a 25 cm^2^ flask. The CRFK cell line was provided by Istituto Zooprofilattico Sperimentale della Lombardia e dell’Emilia (Brescia), Laboratorio Centro Substrati Cellulari were grown in MEM in the presence of 10% fetal calf serum (FCS) and 1% penicillin–streptomycin. Cells were infected at the mitotic phase. At 48 h post-infection (p.i.), typical parvovirus CPE was visible in the flask infected with ileum samples: Cells appeared shrunken, elongated, rounded, and detached. At 72 h p.i., CPE was even more pronounced, with the cells appearing mostly detached and rounded (Figure 7). Cells and medium were collected and centrifuged at 8000 rpm for 10 min. The supernatant was collected and stored at −80 °C, and the cell pellet was stored at −20 °C for nucleic acid extraction.

#### 2.3.2. Molecular Analyses

DNA and RNA were extracted from a portion of the spleen, lung, ileum, and colon of cats #1 and #2 using the DNeasy Blood and Tissue Kit and RNeasy Plus Mini Kits (Qiagen, Hilden, Germany), respectively, according to the manufacturer’s instructions. 

Molecular analyses were performed on all samples to detect Protoparvovirus carnivoran1 and Bocaparvovirus spp, using the HotStarTaq^®^ Plus Master Mix Kit (Qiagen, Germany), and *Coronavirus* spp., *Astrovirus* spp., and *Rotavirus*, using the OneStep RT-PCR Kit (Qiagen, Germany). 

The primer sets and PCR conditions used in this study are summarized in Table 2. For Protoparvovirus carnivoran1, the diagnostic protocol of Schatzberg and colleagues was applied, using three primer pairs amplifying genes encoding structural proteins (VP1 and VP2) of both FPV and CPV [17]. The ileum samples of cats #1 and #2 were positive for Protoparvovirus carnivoran1 using primer pairs 1 and 2. The positivity was confirmed using Sanger sequencing (BMR genomics, Padova, Italy) and BLAST analysis, which reported a close matching with the feline panleukopenia virus. All samples tested negative for the other viruses.

#### 2.3.3. Phylogenetic Analysis

To achieve a better strain characterization, the sequencing of longer genomic regions was attempted on the viral isolates. The pellet from cells showing CPE (see Section 2.3.1) was then used for DNA extraction using the DNeasy Blood and Tissue Kit (Qiagen, Germany). The presence of Protoparvovirus carnivoran1was confirmed using the same protocol previously mentioned. PCRs were performed on the DNA extracted from infected cells using the HotStarTaq^®^ Plus Master Mix Kit (Qiagen, Germany). The complete NS1 and VP portions of the FPV genome were amplified and sequenced. Primer sets are summarized in Table 3. The amplicons were subjected to Sanger sequencing (BMR genomics, Padova, Italy).

Sequences were visually inspected for a quality check using BioEdit [27], and consensus sequences were assembled with Chromas Pro 2.1.8 (Technelysium Pty Ltd., South Brisbane, Australia). Two separate datasets of international reference sequences were downloaded from GenBank for VP1 and NS1 genes (Supplementary Figures S1 and S2). The VP1 and NS1 sequences (Acc. Num. OQ718429) were aligned to the respective reference datasets using MAFFT and trimmed using MEGA X [28,29]. Phylogenetic trees were reconstructed using a maximum likelihood (ML) approach implemented in MEGA X, choosing a substitution model based on the lowest Bayesian information criterion (BIC). Branch support was calculated by performing 1000 bootstrap replicates, and bootstrap values ≥70% were considered reliable.

**Figure 8:**
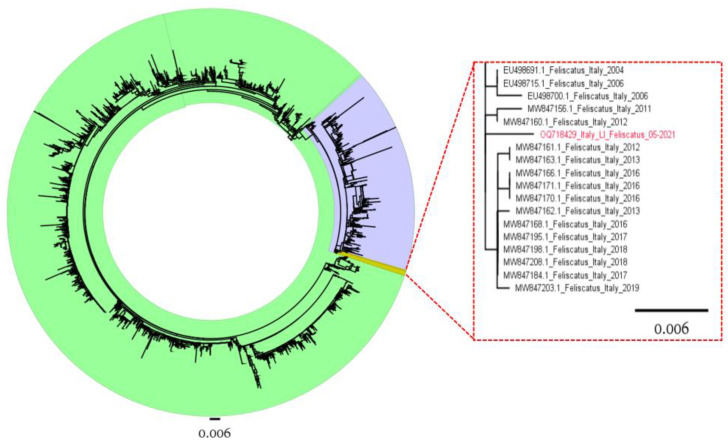
Maximum likelihood phylogenetic tree based on the complete VP2 sequence of the reference dataset. CPV and FPV strains are colored green and blue. In the right insert, the clade comprising the strain isolated in the present study (highlighted in red) is magnified.

Regardless of the considered dataset (NS1, VP1, or VP2), the isolated strain clustered with FPV strains. The analysis was repeated on the VP2 dataset benefitting from the larger sequence availability, which demonstrated its clustering with other Italian strains collected from cats between 2004 and 2019 (Figure 8).

## 3. Discussion

The feline panleukopenia virus (FPV) is a highly contagious pathogen that is mainly transmitted via direct contact with infected cats. FPV causes feline panleukopenia, a disease characterized by a severe reduction in the white blood cell count, immunosuppression, and severe gastroenteritis with degeneration of the intestinal villi and consequent impaired nutrient absorption [3,7,30]. The outcome ranges from subclinical to peracute infection, but the most common is the acute form, which initially presents with specific signs, such as fever, depression, and anorexia, commonly followed by vomiting unrelated to feeding and watery-to-hemorrhagic diarrhea [2]. Because FPV has a predilection for replication in rapidly dividing cells, kittens are most susceptible to infection, but cats of all ages are at risk, especially if they are unvaccinated and live in high-density areas such as animal shelters [9,31]. Although vaccination is considered essential according to the World Small Animal Veterinary Association (WSAVA) [10], many owners do not follow the recommendations, and although there may be good immunity in the domestic cat population, cases of parvovirus are common, indicating that the pathogen is still circulating [2,31,32,33]. 

In this report, we describe a severe outbreak in a group of cohabiting unvaccinated adult cats. The cats lived in the countryside, and although domestic, they had an outdoor life, roaming the surrounding area and feeding outside the house. The clinical course of the outbreak was peracute, with a hemorrhagic pattern, 100% of lethality, and rapid spread. Signs and lesions showed a hemorrhagic pattern of the disease and were consistent with disseminated intravascular coagulation. Secondary bacterial sepsis can be hypothesized following hyperacute FPV infection in unvaccinated cats. The observed clinical–pathological pattern affecting adult cats was quite peculiar since a marked hemorrhagic pattern, instead of lymphoid tissue depletion, villous atrophy, and small intestinal crypt epithelial cell necrosis, was observed [3]. Nevertheless, molecular studies did not highlight peculiar genomic features of the parvovirus isolate. In fact, in the phylogenetic tree reconstructed on the highly variable VP2 region, the strain obtained from cat #1 appeared to cluster with the strains previously identified in Italy (Figure 6). No peculiar amino acid substitutions were identified, lessening the role of the viral phenotype in the abnormal pathogenicity.

The outbreak affected 3 out of 12 cats in a very short time. However, the prompt application of biosecurity measures and vaccination could have resulted in an effective interruption of virus spread. The severity of the disease did not correlate with any known genetic predisposition, as the cats belonged to different genetic lines. Virus isolation and subsequent molecular analysis identified a parvovirus, which was most likely the principal agent responsible for the outbreak, but we cannot exclude the presence of coinfections that could have synergistically increased the pathogenicity of FPV. Although the cats tested negative for the main viruses responsible for enteritis (coronavirus, rotavirus, astrovirus, and bocavirus) during molecular analysis, the involvement of an uninvestigated virus is possible, also considering the potential contacts of the study population with wild animals. In the province of Leghorn, wildlife is abundant and tends to approach inhabited centers in search of food. The outdoor life of the cats increased the likelihood of contact with local wildlife, either directly or indirectly. No other domestic animals had been introduced into the house in the recent past, so wild animals could be considered as the initial source of the outbreak both because of the possibility of sharing outdoor areas of the house and also possible contact with food and water available to the domestic cats. Protoparvovirus carnivoran1also infects wild mammals of the families *Felidae*, *Mustelidae*, *Procyonidae*, and *Viverridae* (including raccoons, ring-tailed cats, foxes, and minks). Virus exchange between the domestic and wild populations has been proven, particularly in overlapping/shared areas [34,35,36,37,38]. Molecular analyses seem to exclude this hypothesis, suggesting a closer relationship of this strain with other strains sampled from the domestic population, but subsampling of wildlife is common, and thus related variants may have been missed. 

Another possibility is that the virus entered the study population indirectly via fomites, considering the high stability of FPV in the environment. 

Remarkably, the POC tests used in the veterinary clinic were negative not only for coronavirus, FIV, and FeLV but also for parvovirus, even when different tests were used. 

This result could be tentatively explained by a dilution effect of the severe hemorrhagic diarrhea, which might have affected the sensitivity of the test. In addition, antigen shedding may be intermittent, limiting the sensitivity of the test, especially if it is performed only once, and it is not the first time that false-negative results have been found [31]. On the other hand, the negative result should not be attributed to poor test inclusivity, as the strain described here does not appear to have any peculiar phenotypical features that might have hindered the detection.

In conclusion, because the infected cats lived closely together, sharing areas inside and outside the house, litter trays, and feeding areas, and they were not vaccinated, we could assume that the virus found the ideal conditions to infect and replicate at high titers, resulting in a particularly aggressive outbreak. 

## Figures and Tables

**Table 1:** Age and status of all cohabiting cats.

Cat No.	Age	Status
1	5	Died few hours after clinical signs
2 (cat #1)	7	Died 24 h after clinical signs; submitted to DSV
3 (cat #2)	5	Died 48 h after clinical signs; submitted to DSV
4	1	I–V
5	1	I–V
6	2	I–V
7	4	I–V
8	5	I–V
9	8	I–V
10	9	I–V
11	11	I–V
12	11	I–V

I–V: Isolated, vaccinated, and without clinical signs.

**Table 2:** Primer set and PCR conditions used in this study for virological investigation.

Viral Target	Primers Sequences		Melting T° (°C)	Amplicon Length (bps)	Reference
Protoparvovirus carnivoran1	Pair 1	Fw: ACGTGGTGTAACTCAAATGG Rw: GCATTTGGTAGACAACATGGT	55	215	[13]
	Pair 2	Fw: GGGTGTGTTAGTAAAGTGGG Rw: CGCTGCTTATCTTCGCTCTG		193	
	Pair 3	Fw: CAAACAAATAGAGCATTGGGC Rw: GCTGAGGTTGGTTATAGTGCACC		184	
Bocaparvovirus	Fw: GCCAGCACNGGNAARACMAA Rw: CATNAGNCAYTCYTCCCACCA		55	141	[21]
Coronavirus	Fw: GGKTGGGAYTAYCCKAARTG Rw: TGYTGTSWRCARAAYTCRTG Fw nested: GGTTGGGACTATCCTAAGTGTGA Rw nested: CCATCATCAGATAGAATCATCAT		48	440	[22]
Astrovirus	Fw a: GARTTYGATTGGRCKCGKTAYGA Fw b: GARTTYGATTGGRCKAGGTAYGA Fw nested a: CGKTAYGATGGKACKATHCC Fw nested b: AGGTAYGATGGKACKATHCC Rw: GGYTTKACCCACATNCCRAA		50	422	[23]
Rotavirus	Fw: GACGGVGCRACTACATGGT Rw: GTCCAATTCATNCCTGGTGG5		55	379	[24]

**Table 3:** Primers used in this study for phylogenetic analysis.

Primer Name	Sequence 5′-3′	Position	Reference
NS_Fext	GACCGTTACTGACATTCGCTTC	206–227	[25]
NS_Fint	GTTGAAACCACAGTGACGACAG	1055–1076	
NS_Rext	GGAGAACCAACTAACCCTTC	2460–2441	
NS_Rint	CACCTGAAGACTGGATGATG	1186–1167	
2161_F	TTGGCGTTACTCACAAAGACGTGC	2161–2184	
4823_R	GTTGTTATGGTGTGGGTGGTTGGT	4823–4800	
VP1_Seq_F2	GGATTTCTACGGGTACTTTC	2713–2732	[26]
VP1_Seq_F3	AGGTGATGAATTTGCTACAGG	3368–3388	
VP1_Seq_F4	GCTACCAACAGATCCAATTG	3887–3906	
VP1_Seq_R2	CTCAGCCACCAACTAAAGTTT	3090–3070	

## Data Availability

The data presented in this study are available on request from the corresponding author.

## Supplementary Materials

The following supporting information can be downloaded at: https://www.mdpi.com/article/10.3390/pathogens12060822/s1, Figure S1: Phylogenetic tree reconstructed on the NS1 gene of available Protoparvovirus carnivoran1 strains downloaded from Genbank.; Figure S2: Phylogenetic tree reconstructed on the VP1 gene of available Protoparvovirus carnivoran1 strains downloaded from Genbank.
