# Supplementary figures and images for: Feline Parvovirus Lethal Outbreak in a Group of Adult Cohabiting Domestic Cats
Source: Pathogens. 2023 Jun 11;12(6):822. doi: 10.3390/pathogens12060822 (PMC10302906; doi:10.3390/pathogens12060822)

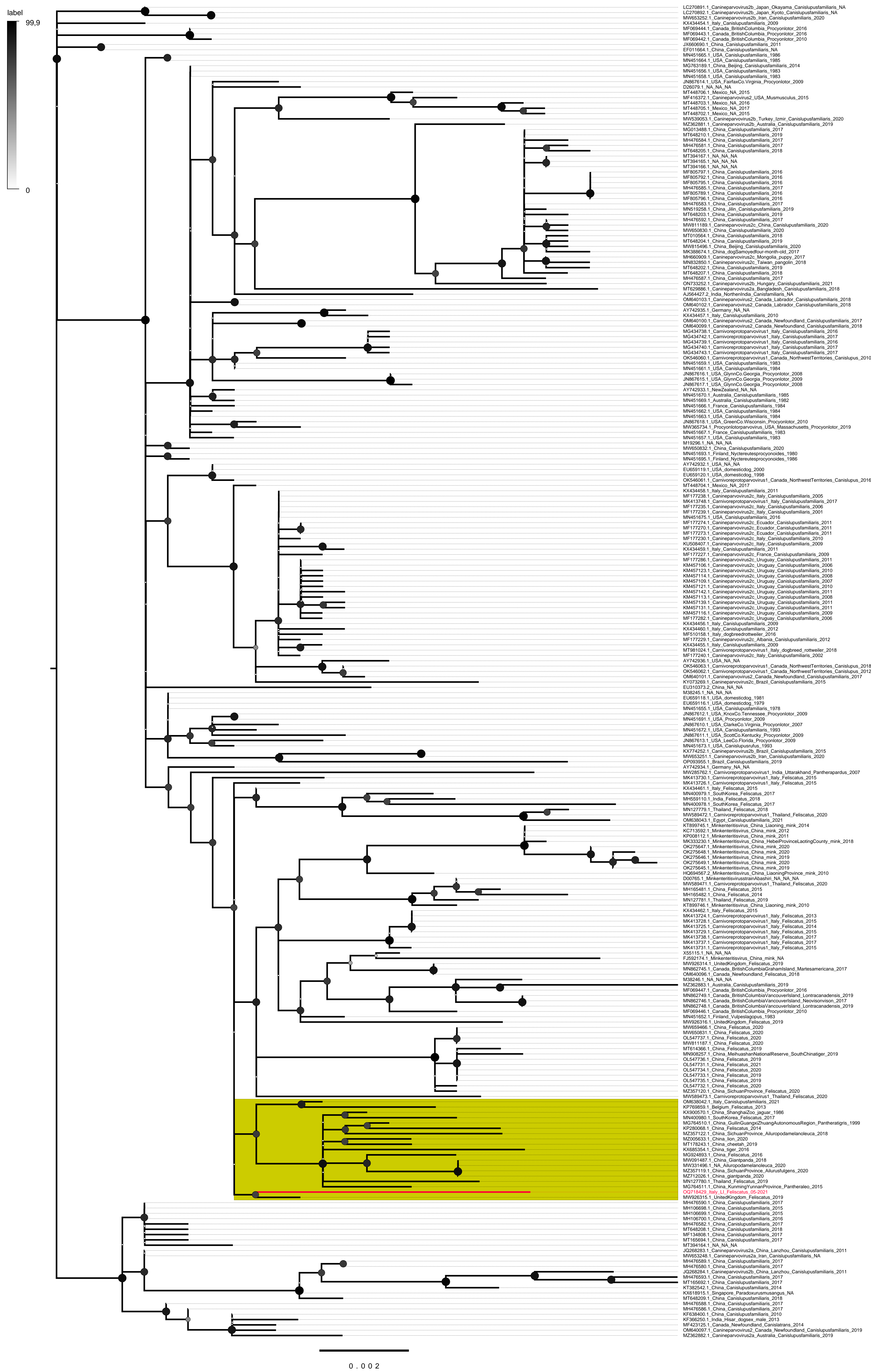

Supplement: Supplementary file 1 [file pathogens-12-00822-s001.zip › FIG S1_NS1_Tree_expanded.pdf]

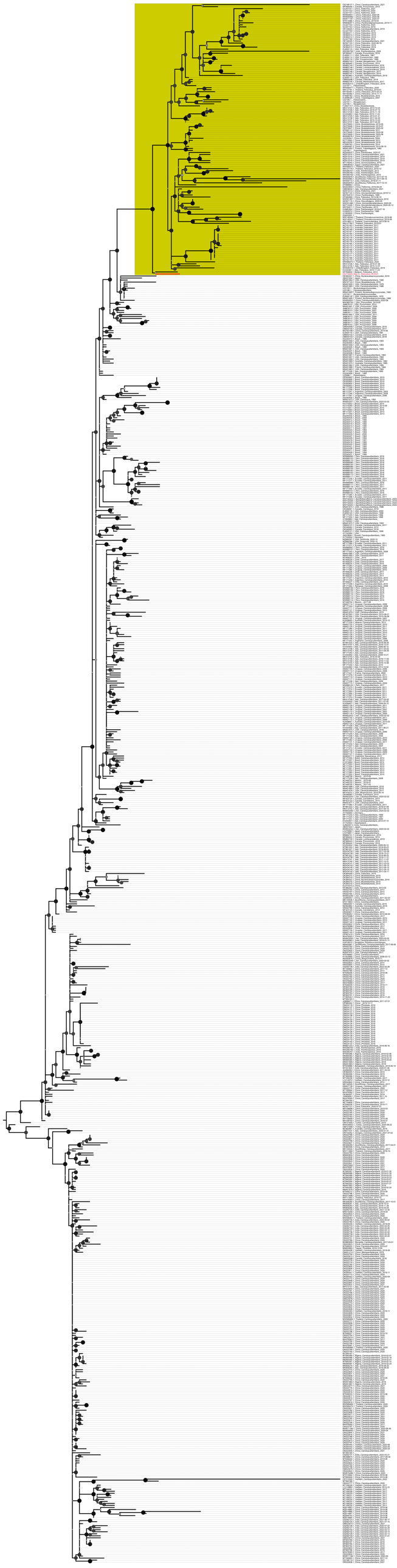

Supplement: Supplementary file 1 [file pathogens-12-00822-s001.zip › FIGS2_Tree_VP1_esteso.pdf]
